# Magnetic resonance imaging of brain structural and functional changes in cognitive impairment associated with Parkinson’s disease

**DOI:** 10.3389/fnagi.2024.1494385

**Published:** 2024-12-04

**Authors:** Danna Cao, Jinhuan Yue, Zeyi Wei, Dong-Hong Huang, Xuchen Sun, Ke-Xuan Liu, Peng Wang, Fan Jiang, Xiaoling Li, Qinhong Zhang

**Affiliations:** ^1^Division of CT and MRI, First Affiliated Hospital of Heilongjiang University of Chinese Medicine, Harbin, China; ^2^Shenzhen Frontiers in Chinese Medicine Research Co., Ltd., Shenzhen, China; ^3^Vitality University, Hayward, CA, United States; ^4^Second Affiliated Hospital of Heilongjiang University of Chinese Medicine, Harbin, China; ^5^First School of Clinical Medicine, Heilongjiang University of Chinese Medicine, Harbin, China; ^6^Department of Radiology, The 962 Hospital Cadre Ward of the Joint Service Support Unit of the Chinese People's Liberation Army, Harbin, China; ^7^Hebei University Health Science Center, Baoding, China; ^8^Division of Oncology, First Affiliated Hospital of Heilongjiang University of Chinese Medicine, Harbin, China; ^9^The Affiliated Hospital of Jiangxi University of Chinese Medicine, Nanchang, China; ^10^Heilongjiang University of Chinese Medicine, Harbin, China

**Keywords:** Parkinson’s disease, cognitive impairment, magnetic resonance imaging, brain structure, brain function

## Abstract

Cognitive impairment is a critical non-motor symptom of Parkinson’s Disease (PD) that profoundly affects patients’ quality of life. Magnetic Resonance Imaging (MRI) has emerged as a valuable tool for investigating the structural and functional brain changes associated with cognitive impairment in PD (PD-CI). MRI techniques enable the precise identification and monitoring of the onset and progression of cognitive deficits in PD. This review synthesizes recent literature on the use of MRI-based techniques, including voxel-based morphometry, diffusion tensor imaging, and functional MRI, in the study of PD-CI. By examining these imaging modalities, the article aims to elucidate the patterns of brain structural and functional alterations in PD-CI, offering critical insights that can inform clinical management and therapeutic strategies. In particular, this review provides a novel synthesis of recent advancements in understanding how specific MRI metrics, such as amplitude of low-frequency fluctuations, regional homogeneity, and functional connectivity, contribute to early detection and personalized treatment approaches for PD-CI. The integration of findings from these studies enhances our understanding of the neural mechanisms underlying cognitive impairment in PD and highlights the potential of MRI as a supportive tool in the clinical assessment and treatment of PD-CI.

## 1 Introduction

Parkinson’s Disease (PD) is a neurodegenerative disorder characterized by rapid progression and a significant increase in incidence and prevalence over the past two decades (Nemade et al., 2021; Dorsey and Bloem, 2018). The risk of PD escalates with age, and it is more commonly observed in males compared to females, with a male-to-female ratio of approximately 1.4:1. While approximately 5–10% of PD cases are monogenic and follow Mendelian inheritance patterns, the majority are sporadic, with unclear etiology potentially involving a complex interplay of genetic and environmental risk factors (Deng et al., 2018; Kouli et al., 2018; Kalinderi et al., 2016).

Cognitive impairment (CI) is a frequent non-motor manifestation of PD, affecting a considerable proportion of patients. Research indicates that the prevalence of cognitive impairment in PD patients is approximately six times higher than that observed in the general population (Suo et al., 2022; Jurcau and Nunkoo, 2021). CI in PD can be categorized into subjective cognitive decline, mild cognitive impairment specific to PD (PD-MCI), and Parkinson’s Disease Dementia (PDD) based on its severity (Aarsland et al., 2021). It is reported that about 26% of PD patients exhibit PD-MCI, with prevalence rates rising to 55% among those with a disease duration of over 10 years (Litvan et al., 2012; Domellöf et al., 2015; Wood et al., 2016). Furthermore, longitudinal studies have shown that 59% of PD-MCI patients progress to dementia within 1 year, highlighting the heightened risk of dementia associated with persistent cognitive impairment in PD (Aarsland et al., 2010; Janvin et al., 2003; Pedersen et al., 2017). Cognitive impairment significantly affects the quality of life of individuals with PD, emphasizing the critical need for early detection and prediction of its progression to improve clinical outcomes (Vasconcellos et al., 2019).

**Figure 1:**
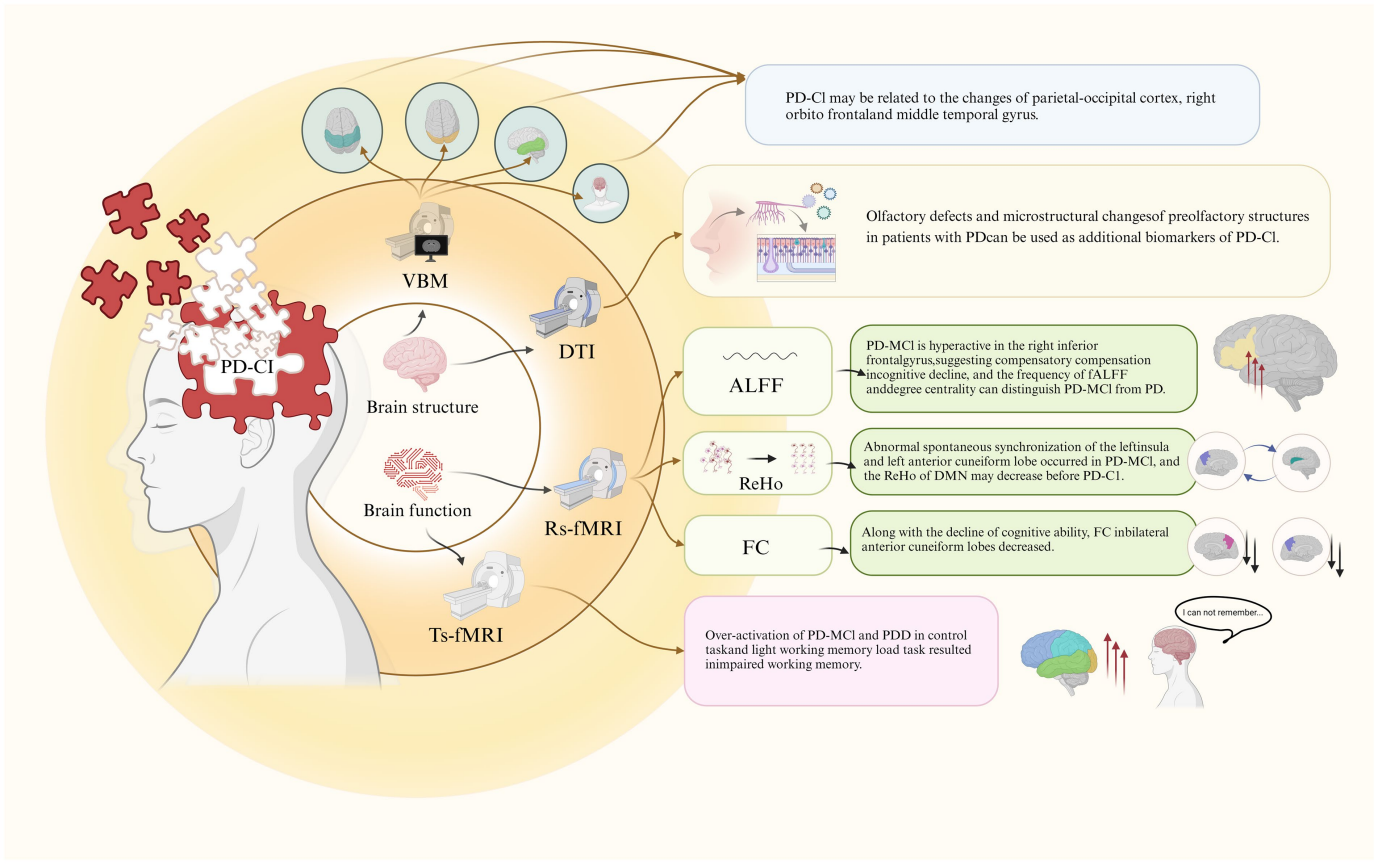
FMRI of brain structure and function in PD-C1.

Magnetic resonance imaging (MRI) is a non-invasive imaging modality widely used in neuroscience to evaluate brain structure, function, and neurochemistry (Achard and Bullmore, 2007). Through MRI, researchers can correlate structural and functional brain measurements with behavioral outcomes or clinical symptoms, offering insights into the potential neural mechanisms underlying various clinical presentations of PD (Brown et al., 2016). MRI plays a crucial role in clinical settings by enhancing the accuracy of diagnosing PD and differentiating its subtypes (Heim et al., 2017). Advanced MRI techniques, including Voxel-Based Morphometry (VBM), Diffusion Tensor Imaging (DTI), and Functional MRI (fMRI), are employed to identify and monitor changes in brain structure and function in patients with PD-CI (Figure 1). These imaging methods provide valuable information that aids in the early identification of cognitive impairment in PD and contributes to the development of targeted therapeutic interventions. By utilizing these advanced MRI techniques, researchers and clinicians can deepen their understanding of the neural underpinnings of cognitive impairment in PD, ultimately supporting improved diagnostic and treatment strategies.

## 2 Brain structural changes in PD-CI

### 2.1 Imaging techniques in PD-CI research

VBM and DTI are prominent MRI techniques utilized to assess brain structural changes in neurodegenerative disorders, including PD and its cognitive manifestations such as PDD and mild cognitive impairment (MCI) (Pan et al., 2013; Wu et al., 2018). VBM quantifies gray matter volume and morphology, while DTI provides insights into white matter microstructural integrity, making them essential tools for exploring the neuropathological underpinnings of PD-CI.

VBM enables the quantitative analysis of gray matter volume, providing detailed insights into localized cortical atrophy in PD-CI (Wu et al., 2018). Its strength lies in its capacity to precisely identify structural abnormalities related to cognitive decline. However, VBM results may be influenced by the inherent variability in image preprocessing and normalization protocols, which can affect cross-study comparisons (Wu et al., 2018). Additionally, VBM is limited in its ability to investigate white matter changes, thus providing an incomplete picture of the neural degeneration in PD-CI (Wu et al., 2018).

DTI offers valuable insights into white matter microstructure, which is often compromised in PD-CI (Zhang et al., 2015). Its ability to detect subtle microstructural changes, even in the absence of significant gray matter atrophy, makes DTI particularly useful for identifying early markers of cognitive decline (Agosta et al., 2014). However, the technique’s sensitivity to motion artifacts and its limitations in resolving complex fiber architecture, such as in areas of crossing fibers, can complicate data interpretation. Despite these limitations, DTI serves as a critical complement to VBM, providing a fuller understanding of the structural underpinnings of PD-CI.

### 2.2 Gray matter changes in PD and PD-MCI

Zhang et al. (2015) applied VBM to compare gray matter volumes between PD patients with and without MCI and healthy controls. The study identified extensive cortical atrophy in PD patients relative to controls, particularly in the temporal, occipital, parietal, and frontal lobes, as well as the right insular cortex, cerebellar posterior lobe, and left caudate nucleus. In PD-MCI patients, significant gray matter reduction was observed in regions including the bilateral cingulate gyrus, lingual gyrus, left anterior cingulate cortex, insula, right superior temporal gyrus, orbitofrontal cortex, central gyrus, and precuneus, highlighting these areas as potential contributors to cognitive decline in PD.

### 2.3 Specific Gray matter alterations in PD-MCI

Among hospitalized PD patients, those with MCI exhibited further gray matter reduction in the anterior cingulate gyrus, middle temporal gyrus, right precuneus, orbitofrontal cortex, and left cingulate gyrus compared to PD patients without MCI. These findings suggest a pattern of cortical atrophy that may underlie cognitive impairment in PD, particularly implicating the temporoparietal association cortex, right orbitofrontal cortex, and middle temporal gyrus as critical regions involved in the cognitive deficits observed in PD-MCI.

### 2.4 White matter integrity and olfactory dysfunction in PD-MCI

Stewart et al. (2023) investigated the association between cognitive impairment, olfactory dysfunction, and white matter integrity in early PD patients using DTI. The study focused on the anterior olfactory structures (AOS), finding that PD-MCI patients had significantly poorer olfactory function and exhibited abnormalities across all DTI metrics in the AOS compared to PD patients without cognitive impairment. These results imply that olfactory deficits and disrupted white matter integrity in olfactory regions could serve as early biomarkers for cognitive decline in PD-MCI (Figure 1).

### 2.5 Implications for PD-CI pathogenesis

The combined findings from these studies underscore the role of widespread gray matter atrophy and white matter microstructural changes as key features of cognitive impairment in PD. The identification of specific brain regions associated with these changes enhances our understanding of the neural substrates of PD-CI and suggests potential targets for future research and therapeutic intervention. Further exploration of these neuroimaging biomarkers could contribute to earlier diagnosis and more personalized management strategies for cognitive impairment in PD.

## 3 Brain functional changes in PD-CI

### 3.1 Overview of fMRI techniques in PD-CI

FMRI is a crucial tool for investigating the brain functional alterations associated with PD-CI (Baggio et al., 2014). Two primary fMRI modalities are commonly employed in research: resting-state fMRI (rs-fMRI) and task-based fMRI (ts-fMRI). Each modality offers distinct insights into the neural mechanisms underlying cognitive decline in PD, making them complementary methods in understanding PD-CI.

VBM is an imaging analysis technique that quantifies differences in brain anatomy by measuring gray matter volume across individuals (Sivaranjini and Sujatha, 2024). It is particularly effective in identifying structural changes, such as atrophy in brain regions, in patients with neurodegenerative diseases, including PD-CI (Sivaranjini and Sujatha, 2024).

DTI is another MRI technique that assesses the diffusion of water molecules in brain tissue, allowing for the evaluation of white matter integrity (Wang et al., 2020). This method is valuable for detecting subtle changes in white matter, which are often associated with cognitive decline in PD (Wang et al., 2020).

In the context of functional brain activity, amplitude of low-frequency fluctuations (ALFF) measures the amplitude of spontaneous low-frequency fluctuations during rs-fMRI (Wang et al., 2018). This analysis detects regional brain activity changes, which are particularly relevant in assessing cognitive impairments in PD-CI (Wang et al., 2018).

Regional homogeneity (ReHo) evaluates the synchronization of brain activity across neighboring voxels, providing insights into local brain connectivity. Alterations in ReHo can signal disruptions in functional networks, which are commonly observed in PD-CI (Li et al., 2020).

Functional connectivity (FC) assesses the temporal correlation between spatially distant brain regions, highlighting how different parts of the brain communicate. FC analysis is particularly useful in identifying network disruptions, a hallmark of PD-CI (Amboni et al., 2015).

Rs-fMRI captures spontaneous brain activity while the subject is at rest, relying on blood oxygen level-dependent (BOLD) signal fluctuations to assess intrinsic brain networks like the default mode network (DMN) (Day et al., 2019). Rs-fMRI is highly effective for detecting disruptions in FC within these networks, which are often implicated in PD-CI. Additionally, rs-fMRI can identify compensatory mechanisms, where increased activity in specific regions reflects the brain’s attempts to counterbalance cognitive deficits (Kaut et al., 2020). Since rs-fMRI does not rely on task performance, it is particularly sensitive to early functional changes that might go undetected during cognitive tasks.

On the other hand, ts-fMRI examines brain activation patterns in response to specific cognitive tasks or stimuli. It provides a more targeted approach, allowing researchers to observe how task-related brain regions function and how these processes deteriorate in PD-CI (Baggio and Junqué, 2019). Ts-fMRI is particularly useful for isolating the neural dynamics related to specific cognitive functions, such as memory or attention. While both rs-fMRI and ts-fMRI can detect compensatory mechanisms, rs-fMRI tends to be more sensitive to early intrinsic changes since it captures spontaneous brain activity without the need for external stimuli (Baggio and Junqué, 2019).

Key analysis methods used in rs-fMRI include ALFF, ReHo, and FC. These metrics help provide a comprehensive view of how brain regions synchronize and communicate, thus revealing detailed patterns of how functional networks are disrupted in PD-CI (Sun et al., 2021; Ten Kate et al., 2018; Hao et al., 2022; Li et al., 2021) (Figure 1). In contrast, ts-fMRI focuses on task-induced activations, which helps pinpoint which specific brain functions are compromised by PD (Kang et al., 2021) (Figure 1).

Despite the valuable insights both modalities offer, they have limitations. Rs-fMRI excels in detecting intrinsic network dysfunction without external stimuli, making it particularly useful for identifying early cognitive impairments (Lemée et al., 2019). Ts-fMRI, however, provides a focused analysis of brain activity during specific tasks. Both modalities face challenges, such as fMRI’s relatively low spatial resolution, which may obscure subtle neural activity (Smith et al., 2011). Moreover, they are susceptible to artifacts from head motion and physiological noise, potentially reducing the reliability of connectivity measurements (Smith et al., 2011). Additionally, interpreting fMRI data is complex due to the intricate interactions between various brain regions and networks (Smith et al., 2011).

Nevertheless, fMRI remains an indispensable tool for understanding the dynamic functional changes that accompany cognitive decline in PD. It provides valuable insights into the underlying neural mechanisms of PD-CI, supporting ongoing advancements in research and clinical practice.

### 3.2 Resting-state functional changes

#### 3.2.1 ALFF findings in PD-MCI

Wang et al. (2018) employed ALFF to measure spontaneous neural activity across PD patients with normal cognition (PD-NC), PD-MCI, and healthy controls (HC). Both PD groups exhibited reduced ALFF in the occipital regions (Calcarine_R/Cuneus_R) compared to HC, suggesting altered basal neural activity. Notably, PD-MCI patients demonstrated increased ALFF in the right inferior frontal gyrus (Frontal_Inf_Oper_R) relative to HC and PD-NC, potentially indicating compensatory hyperactivity linked to cognitive challenges. A positive correlation between ALFF in Frontal_Inf_Oper_R and Unified Parkinson’s Disease Rating Scale scores, alongside a weak negative correlation with the Montreal Cognitive Assessment (MoCA) score, suggests this hyperactivity may reflect early compensatory mechanisms in PD-MCI.

#### 3.2.2 Frequency-dependent neural activity alterations

Rong et al. (2021) explored frequency-specific neural activity changes using fractional ALFF (fALFF) and degree centrality (DC) metrics in PD-MCI. The study found that these measures varied with specific frequency bands, revealing distinct patterns of disruption that differentiated PD-MCI from PD-NC. These results suggest that frequency-dependent abnormalities in neural activity may serve as useful biomarkers for identifying and characterizing cognitive impairment in PD.

#### 3.2.3 ReHo findings in PD-MCI

Li et al. (2020) investigated ReHo, a measure of local synchronization of brain activity, in PD patients using rs-fMRI. The study found that compared to HC, PD patients showed reduced ReHo in the left posterior cerebellum, while PD-MCI patients had increased ReHo in the marginal lobes and bilateral precuneus/left superior parietal lobule, along with decreased ReHo in the left insula. The negative correlation between ReHo in the left precuneus and cognitive scores, along with a positive correlation in the left insula, indicates that PD-MCI is associated with disrupted local synchronization, particularly in regions implicated in cognitive processing.

### 3.3 Task-state functional changes

#### 3.3.1 Alterations in ReHo and the default mode network (DMN)

A study by Xing et al. (2021) assessed ReHo changes in PD-CI relative to PD-NC and HC, identifying elevated ReHo in the right middle frontal gyrus (MFG) and reduced ReHo in the left precuneus, bilateral inferior parietal lobule (IPL), and right posterior cingulate gyrus (PCG) in PD-NC compared to HC. PD-MCI patients exhibited increased ReHo in the right PCG, left middle occipital gyrus (MOG), and IPL. These findings suggest that reduced ReHo within the DMN may precede cognitive impairment onset, with compensatory increases in ReHo in areas such as the right MFG potentially reflecting adaptive responses to early cognitive decline.

#### 3.3.2 FC alterations in cognitive networks

Wang et al. (2021) explored FC changes in the posterior cingulate cortex (PCC), a key node of the DMN, among PD-MCI patients. Compared to PD-NC and HC, PD-MCI patients showed reduced FC in the bilateral precuneus, a region critically involved in cognitive functions. The decreased FC was associated with poorer cognitive performance, underscoring the role of the precuneus and broader DMN disruptions in the pathogenesis of cognitive impairment in PD.

#### 3.3.3 Compensatory mechanisms in working memory tasks

Hattori et al. (2022) investigated neural responses during working memory (WM) tasks across PD patients with varying cognitive statuses. The study found that PD patients with normal cognition maintained WM performance through excessive activation in moderate and high WM load tasks, indicating compensatory neural mechanisms. In contrast, PD-MCI and PDD patients exhibited excessive activation even during low WM load tasks, suggesting a depletion of neural resources and impaired WM performance. Key brain regions implicated included the bilateral dorsolateral prefrontal cortex, frontal eye field, inferior/superior parietal lobules, and caudate nucleus, reflecting the differential recruitment of neural circuits depending on cognitive status and task demands.

## 4 Limitations and future directions

Despite the advancements in understanding the neural correlates of PD-CI using MRI techniques, several limitations remain in current research.

### 4.1 Need for longitudinal studies

A significant limitation is the lack of longitudinal studies that compare different stages of PD-CI. Such studies are crucial for understanding the progression of cognitive impairment over time and how it may be influenced by compensatory mechanisms or the effects of treatment. Future research should aim to include longitudinal designs to better capture the dynamic changes in brain structure and function associated with the progression of PD-CI. Specifically, studies could focus on tracking individual patients over several years, monitoring how their brain connectivity patterns change, and identifying early biomarkers for cognitive decline. Additionally, longitudinal imaging studies combined with cognitive assessments may help differentiate the trajectories of MCI from those leading to dementia, thus informing personalized therapeutic strategies.

### 4.2 Limited task-state fMRI research

There is a notable scarcity of ts-fMRI studies focused on PD-CI conducted by both domestic and international researchers. Ts-fMRI is valuable for assessing brain activation patterns during specific cognitive tasks, yet its application in PD-CI remains underexplored. Future studies should prioritize ts-fMRI experiments, particularly those examining the effects of cognitive tasks, attention, and motor training, to provide a more comprehensive understanding of functional alterations in PD-CI. A potential direction would be to design ts-fMRI protocols that target specific cognitive domains, such as working memory, executive function, and visuospatial abilities, which are frequently impaired in PD-CI. Comparing brain activation patterns across different cognitive loads could reveal the compensatory mechanisms engaged by PD-CI patients and inform tailored interventions that strengthen cognitive resilience.

### 4.3 Variation in MRI techniques

Different MRI techniques, such as VBM, DTI, rs-fMRI, and ts-fMRI, each offer unique insights into brain structure and function in PD-CI. However, these techniques emphasize different aspects of brain alterations, and there is currently no consensus on which single technique or combination of techniques provides the most accurate predictive value for cognitive impairment in PD. Future research should focus on systematically comparing these techniques or exploring integrated approaches to identify the most effective diagnostic and prognostic tools for PD-CI. Multimodal imaging studies that combine structural and functional MRI data could provide more comprehensive biomarkers for early cognitive decline in PD. Moreover, machine learning algorithms could be employed to integrate data from multiple modalities and enhance diagnostic accuracy.

### 4.4 Potential for advancements in MRI technology

As MRI technology continues to evolve, there is significant potential for further advancements in the diagnosis and treatment of PD-CI. Enhanced imaging resolution, more sophisticated analysis methods, and the development of multimodal imaging approaches could provide deeper insights into the neurobiological mechanisms underlying cognitive impairment in PD. Future research should leverage these technological advancements to improve the clinical application of MRI in the management of PD-CI. For example, high-field MRI scanners could offer more detailed structural and functional brain images, allowing for the detection of subtle changes that are currently missed by conventional MRI. Additionally, the integration of MRI with other imaging modalities, such as positron emission tomography, may help identify neurochemical changes alongside structural and functional alterations in the brain.

Addressing these limitations will be critical for advancing our understanding of PD-CI and improving diagnostic and therapeutic strategies through the continued development of MRI technology.

## 5 Summary

In summary, the use of MRI technology has significantly advanced the understanding of brain structural and functional alterations in patients with PD-CI. VBM has been instrumental in identifying gray matter atrophy in brain regions that are closely associated with cognitive decline, such as the temporal and frontal lobes, cingulate cortex, and insula. DTI has provided insights into microstructural changes in white matter tracts, revealing disruptions that may contribute to cognitive deficits in PD-CI. Rs-fMRI studies have shown altered functional connectivity and activity in key brain regions including the frontal gyrus, cingulate gyrus, insula, precentral gyrus, precuneus, and inferior parietal lobule, highlighting changes in the intrinsic functional networks of the brain. Ts-fMRI has demonstrated excessive activation in neural circuits during working memory tasks, suggesting compensatory mechanisms or neural inefficiencies in PD-CI patients. Collectively, these MRI findings enhance the prediction and diagnosis of cognitive impairment in PD, offering valuable imaging biomarkers that could inform clinical strategies aimed at mitigating disease progression and tailoring therapeutic interventions. Addressing these findings could support the development of more effective diagnostic criteria and treatment plans, ultimately improving patient outcomes in PD-CI.

## References

[ref1] Aarsland D. Batzu L. Halliday G. M. Geurtsen G. J. Ballard C. Ray Chaudhuri K.. (2021). Parkinson disease associated cognitive impairment. Nat. Rev. Dis. Primers 7:47. doi: 10.1038/s41572-021-00280-3 34210995

[ref2] Aarsland D. Bronnick K. Williams-Gray C. Weintraub D. Marder K. Kulisevsky J.. (2010). Mild cognitive impairment in Parkinson disease: a multicenter pooled analysis. Neurology 75, 1062–1069. doi: 10.1212/WNL.0b013e3181f39d0e, PMID: 20855849 PMC2942065

[ref3] Achard S. Bullmore E. (2007). Efficiency and cost of economical brain functional networks. PLoS Comput. Biol. 3:e17. doi: 10.1371/journal.pcbi.0030017, PMID: 17274684 PMC1794324

[ref4] Agosta F. Canu E. Stefanova E. Sarro L. Tomić A. Špica V.. (2014). Mild cognitive impairment in Parkinson's disease is associated with a distributed pattern of brain white matter damage. Hum. Brain Mapp. 35, 1921–1929. doi: 10.1002/hbm.22302 23843285 PMC6869219

[ref5] Amboni M. Tessitore A. Esposito F. Santangelo G. Picillo M. Vitale C.. (2015). Resting-state functional connectivity associated with mild cognitive impairment in Parkinson's disease. J. Neurol. 262, 425–434. doi: 10.1007/s00415-014-7591-5 25428532

[ref6] Baggio H. C. Junqué C. (2019). Functional MRI in Parkinson's disease cognitive impairment. Int. Rev. Neurobiol. 144, 29–58. doi: 10.1016/bs.irn.2018.09.010 30638456

[ref7] Baggio H. C. Sala-Llonch R. Segura B. Marti M. J. Valldeoriola F. Compta Y.. (2014). Functional brain networks and cognitive deficits in Parkinson's disease. Hum. Brain Mapp. 35, 4620–4634. doi: 10.1002/hbm.22499, PMID: 24639411 PMC6869398

[ref8] Brown H. D. Woodall R. L. Kitching R. E. Baseler H. A. Morland A. B. (2016). Using magnetic resonance imaging to assess visual deficits: a review. Ophthalmic Physiol. Opt. 36, 240–265. doi: 10.1111/opo.12293, PMID: 27112223 PMC4855621

[ref9] Day T. K. M. Madhyastha T. M. Lee A. Zabetian C. P. Montine T. J. Grabowski T. J. (2019). Effect of dopaminergic medications on blood oxygen level-dependent variability and functional connectivity in Parkinson's disease and healthy aging. Brain Connect. 9, 554–565. doi: 10.1089/brain.2019.0677, PMID: 31131605 PMC6727479

[ref10] Deng H. Wang P. Jankovic J. (2018). The genetics of Parkinson disease. Ageing Res. Rev. 42, 72–85. doi: 10.1016/j.arr.2017.12.007 29288112

[ref11] Domellöf M. E. Ekman U. Forsgren L. Elgh E. (2015). Cognitive function in the early phase of Parkinson’s disease, a five-year follow-up. Acta Neurol. Scand. 132, 79–88. doi: 10.1111/ane.12375, PMID: 25644230

[ref12] Dorsey E. R. Bloem B. R. (2018). The Parkinson pandemic: a call to action. JAMA Neurol. 75, 9–10. doi: 10.1001/jamaneurol.2017.3299, PMID: 29131880

[ref13] Hao X. Liu Z. He S. Wang Y. Zhao Y. Wang R. (2022). Application of DTI and fMRI in moyamoya disease. Front. Neurol. 13:948830. doi: 10.3389/fneur.2022.948830, PMID: 35989917 PMC9391058

[ref14] Hattori T. Reynolds R. Wiggs E. Horovitz S. G. Lungu C. Chen G.. (2022). Neural correlates of working memory and compensation at different stages of cognitive impairment in Parkinson's disease. Neuroimage Clin. 35:103100. doi: 10.1016/j.nicl.2022.103100, PMID: 35780660 PMC9421432

[ref15] Heim B. Krismer F. De Marzi R. Seppi K. (2017). Magnetic resonance imaging for the diagnosis of Parkinson's disease. J. Neural Transm. (Vienna) 124, 915–964. doi: 10.1007/s00702-017-1717-8, PMID: 28378231 PMC5514207

[ref16] Janvin C. Aarsland D. Larsen J. P. Hugdahl K. (2003). Neuropsychological profile of patients with Parkinson’s disease without dementia. Dement. Geriatr. Cogn. Disord. 15, 126–131. doi: 10.1159/000068483 12584427

[ref17] Jurcau A. Nunkoo V. S. (2021). Clinical markers may identify patients at risk for early Parkinson's disease dementia: a prospective study. Am. J. Alzheimers Dis. Other Dement. 36:15333175211021369. doi: 10.1177/15333175211021369, PMID: 34075800 PMC10624063

[ref18] Kalinderi K. Bostantjopoulou S. Fidani L. (2016). The genetic background of Parkinson's disease: current progress and future prospects. Acta Neurol. Scand. 134, 314–326. doi: 10.1111/ane.12563, PMID: 26869347

[ref19] Kang J. M. Kim N. Lee S. Y. Woo S. K. Park G. Yeon B. K.. (2021). Effect of cognitive training in fully immersive virtual reality on visuospatial function and frontal-occipital functional connectivity in predementia: randomized controlled trial. J. Med. Internet Res. 23:e24526. doi: 10.2196/24526, PMID: 33955835 PMC8138710

[ref20] Kaut O. Mielacher C. Hurlemann R. Wüllner U. (2020). Resting-state fMRI reveals increased functional connectivity in the cerebellum but decreased functional connectivity of the caudate nucleus in Parkinson's disease. Neurol. Res. 42, 62–67. doi: 10.1080/01616412.2019.1709141, PMID: 31900094

[ref21] Kouli A. Torsney K. M. Kuan W. L. (2018). “Parkinson’s disease: etiology, neuropathology, and pathogenesis” in Parkinson’s disease: Pathogenesis and clinical aspects. eds. Stoker T. B. Greenland J. C. (Brisbane, AU: Codon Publications). 30702842

[ref22] Lemée J. M. Berro D. H. Bernard F. Chinier E. Leiber L. M. Menei P.. (2019). Resting-state functional magnetic resonance imaging versus task-based activity for language mapping and correlation with perioperative cortical mapping. Brain Behav. 9:e01362. doi: 10.1002/brb3.1362, PMID: 31568681 PMC6790308

[ref23] Li H. Jia X. Li Y. Jia X. Yang Q. (2021). Aberrant amplitude of low-frequency fluctuation and degree centrality within the default mode network in patients with vascular mild cognitive impairment. Brain Sci. 11:1534. doi: 10.3390/brainsci11111534 34827533 PMC8615791

[ref24] Li M. G. Liu T. F. Zhang T. H. Chen Z. Y. Nie B. B. Lou X.. (2020). Alterations of regional homogeneity in Parkinson's disease with mild cognitive impairment: a preliminary resting-state fMRI study. Neuroradiology 62, 327–334. doi: 10.1007/s00234-019-02333-7, PMID: 31822931

[ref25] Litvan I. Goldman J. G. Tröster A. I. Schmand B. A. Weintraub D. Petersen R. C.. (2012). Diagnostic criteria for mild cognitive impairment in Parkinson’s disease: movement disorder society task force guidelines. Mov. Disord. 27, 349–356. doi: 10.1002/mds.24893, PMID: 22275317 PMC3641655

[ref26] Nemade D. Subramanian T. Shivkumar V. (2021). An update on medical and surgical treatments of Parkinson's disease. Aging Dis. 12, 1021–1035. doi: 10.14336/AD.2020.1225, PMID: 34221546 PMC8219497

[ref27] Pan P. L. Shi H. C. Zhong J. G. Xiao P. R. Shen Y. Wu L. J.. (2013). Gray matter atrophy in Parkinson’s disease with dementia: evidence from meta-analysis of voxel-based morphometry studies. Neurol. Sci. 34, 613–619. doi: 10.1007/s10072-012-1250-3 23184330

[ref28] Pedersen K. F. Larsen J. P. Tysnes O. B. Alves G. (2017). Natural course of mild cognitive impairment in Parkinson disease: a 5-year population-based study. Neurology 88, 767–774. doi: 10.1212/WNL.0000000000003634, PMID: 28108638

[ref29] Rong S. Zhang P. He C. Li Y. Li X. Li R.. (2021). Abnormal neural activity in different frequency bands in Parkinson's disease with mild cognitive impairment. Front. Aging Neurosci. 13:709998. doi: 10.3389/fnagi.2021.709998, PMID: 34489679 PMC8417797

[ref30] Sivaranjini S. Sujatha C. M. (2024). Analysis of cognitive dysfunction in Parkinson's disease using voxel based morphometry and radiomics. Cogn. Process. 25, 521–532. doi: 10.1007/s10339-024-01197-x 38714621

[ref31] Smith S. M. Miller K. L. Salimi-Khorshidi G. Webster M. Beckmann C. F. Nichols T. E.. (2011). Network modelling methods for FMRI. NeuroImage 54, 875–891. doi: 10.1016/j.neuroimage.2010.08.063 20817103

[ref32] Stewart S. A. Pimer L. Fisk J. D. Rusak B. Leslie R. A. Eskes G.. (2023). Olfactory function and diffusion tensor imaging as markers of mild cognitive impairment in early stages of Parkinson's disease. Clin. EEG Neurosci. 54, 91–97. doi: 10.1177/15500594211058263, PMID: 34841903 PMC9693894

[ref33] Sun H. He Y. Cao H. (2021). Functional magnetic resonance imaging research in China. CNS Neurosci. Ther. 27, 1259–1267. doi: 10.1111/cns.13725, PMID: 34492160 PMC8504522

[ref34] Suo X. Lei D. Li N. Peng J. Chen C. Li W.. (2022). Brain functional network abnormalities in Parkinson's disease with mild cognitive impairment. Cereb. Cortex 32, 4857–4868. doi: 10.1093/cercor/bhab520, PMID: 35078209 PMC9923713

[ref35] Ten Kate M. Ingala S. Schwarz A. J. Fox N. C. Chételat G. van Berckel B. N. M.. (2018). Secondary prevention of Alzheimer's dementia: neuroimaging contributions. Alzheimers Res. Ther. 10:112. doi: 10.1186/s13195-018-0438-z, PMID: 30376881 PMC6208183

[ref36] Vasconcellos L. F. R. Pereira J. S. Charchat-Fichman H. Greca D. Cruz M. Blum A. L.. (2019). Mild cognitive impairment in Parkinson's disease: characterization and impact on quality of life according to subtype. Geriatr Gerontol Int 19, 497–502. doi: 10.1111/ggi.13649, PMID: 30912284

[ref37] Wang Q. He W. Liu D. Han B. Jiang Q. Niu J.. (2021). Functional connectivity in Parkinson's disease patients with mild cognitive impairment. Int. J. Gen. Med. 14, 2623–2630. doi: 10.2147/IJGM.S300422 34168488 PMC8218241

[ref38] Wang Z. Jia X. Chen H. Feng T. Wang H. (2018). Abnormal spontaneous brain activity in early Parkinson's disease with mild cognitive impairment: a resting-state fMRI study. Front. Physiol. 9:1093. doi: 10.3389/fphys.2018.01093 30154730 PMC6102476

[ref39] Wang W. Mei M. Gao Y. Huang B. Qiu Y. Zhang Y.. (2020). Changes of brain structural network connection in Parkinson's disease patients with mild cognitive dysfunction: a study based on diffusion tensor imaging. J. Neurol. 267, 933–943. doi: 10.1007/s00415-019-09645-x, PMID: 31792673

[ref40] Wood K. L. Myall D. J. Livingston L. Melzer T. R. Pitcher T. L. MacAskill M. R.. (2016). Different PD-MCI criteria and risk of dementia in Parkinson’s disease: 4-year longitudinal study. NPJ Parkinsons Dis. 2:15027. doi: 10.1038/npjparkd.2015.27, PMID: 28725690 PMC5516585

[ref41] Wu X. Zhang J. Cui Z. Tang W. Shao C. Hu J.. (2018). White matter deficits underlying the impaired consciousness level in patients with disorders of consciousness. Neurosci. Bull. 34, 668–678. doi: 10.1007/s12264-018-0253-3, PMID: 29987517 PMC6060210

[ref42] Xing Y. Fu S. Li M. Ma X. Liu M. Liu X.. (2021). Regional neural activity changes in Parkinson's disease-associated mild cognitive impairment and cognitively normal patients. Neuropsychiatr. Dis. Treat. 17, 2697–2706. doi: 10.2147/NDT.S323127, PMID: 34429605 PMC8380131

[ref43] Zhang Y. Wu I. W. Buckley S. Coffey C. S. Foster E. Mendick S.. (2015). Diffusion tensor imaging of the nigrostriatal fibers in Parkinson's disease. Mov. Disord. 30, 1229–1236. doi: 10.1002/mds.26251, PMID: 25920732 PMC4418199

[ref44] Zhang J. Zhang Y. T. Hu W. D. Li L. Liu G. Y. Bai Y. P. (2015). Gray matter atrophy in patients with Parkinson's disease and those with mild cognitive impairment: a voxel-based morphometry study. Int. J. Clin. Exp. Med. 8, 15383–15392, PMID: 26629027 PMC4658916

